# Caffeinated energy drink consumption among Emirati adolescents is associated with a cluster of poor physical and mental health, and unhealthy dietary and lifestyle behaviors: a cross-sectional study

**DOI:** 10.3389/fpubh.2023.1259109

**Published:** 2023-10-16

**Authors:** MoezAlIslam E. Faris, Fakir Al Gharaibeh, M. Rezaul Islam, Dana Abdelrahim, Eman Rashid Saif, Eman Ali Turki, Mahra Khalfan Al-Kitbi, Salma Abu-Qiyas, Falak Zeb, Hayder Hasan, Mona S. Hashim, Tareq M. Osaili, Hadia Radwan, Leila Cheikh Ismail, Farah Naja, Fatima Zohra Bettayeb, Reyad Shaker Obaid

**Affiliations:** ^1^Department of Clinical Nutrition and Dietetics, College of Health Sciences, University of Sharjah, Sharjah, United Arab Emirates; ^2^Research Institute for Medical and Health Research (RIMHS), University of Sharjah, Sharjah, United Arab Emirates; ^3^Research Institute of Humanities and Social Sciences, University of Sharjah, Sharjah, United Arab Emirates; ^4^Health Promotion Department, Supreme Council for Family Affairs, Sharjah, United Arab Emirates; ^5^Department of Nutrition and Food Technology, Faculty of Agriculture, Jordan University of Science and Technology, Irbid, Jordan

**Keywords:** adolescents, schoolchildren, energy drinks, caffeine, health behaviors, diet, health practice, lifestyle behaviors

## Abstract

**Background:**

Consumption of caffeinated energy drinks (CED) has escalated during the last few years, especially among schoolchildren, with evident adverse health sequelae in this critical age group.

**Objective:**

This study examined the prevalence of CED consumption and its associations with sleep, physical and mental health, and dietary and lifestyle habits among schoolchildren in the United Arab Emirates (UAE).

**Method:**

A structured self-administered online questionnaire was developed and disseminated among schoolchildren aged 14–18 years, selected from schools of the seven emirates of the UAE.

**Results:**

More than 4,500 (*N*= 4,648) responses received. A relatively low prevalence of CED consumption (20%) was found among schoolchildren in the UAE. However, those who reported CED consumption were more likely to report unhealthy dietary (skipping breakfast, frequent snacking, and eating fast foods, low fruit, and vegetable intake) and lifestyle behaviors (long screen time, poor sleep health), in addition to poor self-reported mental and physical health than non-users. CED consumption was significantly and variably associated with multiple sociodemographic factors such as students’ nationality, parental companionship, sex, school type, education level (children’s and parents’), daily allowance, academic performance, screen time, sleep quality parameters, self-reported physical and mental health, and parents’ employment. Sources of knowledge about CED were social media (55%), friends/schoolmates (52%), and family members (52%). Students believed that CED constitute sugar (87%), caffeine (69%), artificial flavors (67%) sweeteners (54%), and stimulating components (43%). The majority (70%) of students reported that CED consumption increases the risks for heart disease, diabetes, high blood sugar (65%), addiction (64%), high blood pressure (59%), and obesity (57%).

**Conclusion:**

These results offer important insights for health professionals, child health specialists, policymakers, and parents in the UAE regarding adolescents’ attitudes, knowledge and behaviors toward CED consumption.

## Introduction

Caffeinated energy drinks (CED) are beverages that contain caffeine and are marketed as enhancing concentration, reducing fatigue, and increasing energy (1). Consumption of CED is alarmingly escalating throughout the globe, with major concern directed toward its increased consumption among schoolchildren and adolescents, which may impose adverse health consequences on this critical age group (2, 3). In the Gulf Corporation Council (GCC) countries, including UAE, consumption of CED is exponentially increasing among children and adolescents (4).

Excessive caffeine intake via products such as CED, coffee, tea, soda, and chocolate bars can impose negative effects on children, including anxiety and poor sleep (5). These adverse effects are ascribed to the inclusion of caffeine, guarana, and taurine in the CED, with supposed performance-enhancing effects; making CED not recommended food for children and adolescents (6, 7). Most recently, Puupponen et al. (8) found that regular intake of CED was linked to inadequate sleep, poor self-rated health, and various health issues. Common reasons for consuming CED include taste/flavor, feeling alert, and getting energy (9). However, many children and adolescents remain unaware of the ingredients of CED, including caffeine, and their health implications (10).

In the United States, consumption rates among schoolchildren were varied and based on age, sex, and ethnicity (11), with a consistent and strong direct relationship between CED consumption and risk-taking behaviors such as alcohol consumption. Such positive associations between CED consumption and alcohol intake, alongside cannabis use, snus use, problematic social media use, short sleep skipping breakfast, drunkenness, and inadequate tooth brushing; were all reported among Finnish schoolchildren (8). Further, the consumption of CED by schoolchildren is also concerning because of its associations with increased risks for aggressive behaviors: truancy, fighting, and bullying (12, 13). Lastly, the high levels of sugar and acid in CED make them erosive beverages that lead to tooth decay and enamel erosion, which can result in long-term dental health problems (14).

The prevalence of CED consumption by school children and adolescents has been a topic of interest during the last decade, with variable prevalence reported in different parts of the world. The prevalence ranged variably from 24.4% in Finland (15), 28.0–31.3% in Hungary (16, 17), about 12–33% in Ontario/Canada (18), 35% in New Zealand (19), 42.3% in the Pacific region (20), 41.4–75.5% in Italy (6, 21, 22), 51.2% in Australia (23), 61.7% in Germany (24), 62% in Atlantic/Canada (25), 63% in Congo (26), and about 66% in the United States (27). In the GCC, the prevalence of CED consumption by schoolchildren reached 45–60% among middle and secondary schoolchildren (28, 29). In the UAE, only one study in Al Ain City/UAE revealed a prevalence of 27% among schoolchildren (30). Considering the large number of schoolchildren in the UAE distributed over the seven emirates, it is crucial to have an accurate estimate of the prevalence of CED consumption among UAE schoolchildren and to examine the relationship with the different health aspects and dietary and lifestyle behaviors.

Due to the improved economic and purchasing power of the schoolchildren and adolescents in the UAE, many types of CED are becoming widely available and affordable, with noticeable and obvious consumption of CED by young schoolchildren seen in different community settings (despite the legal prohibition of selling CED for those who are younger than 16 years old). The parental knowledge and practices, with their educational levels and sociodemographic backgrounds, toward CED are of pivotal significance in exploring the practice of CED consumption among schoolchildren in the UAE. Due to the lack of large-scale national assessment for CED consumption in the UAE, it becomes crucial to examine the prevalence of CED consumption among schoolchildren and young adolescents, to find out its relationship with nutritional status, sleep quality, physical and mental health, and dietary and lifestyle behaviors, and to explore the relationship with parental knowledge and practices toward CED in the UAE.

## Methods

The Strengthening the Reporting of Observational Studies in Epidemiology (STROBE) statement was followed in the planning, implementation, and reporting of the present work (31).

### Study design and description

This study used a cross-sectional design to assess CED consumption among a convenience sample of Schoolchildren across the UAE. Data collection started in November 2021 and concluded in March 2022. Data were collected using an online self-administered questionnaire disseminated via Google Forms. The questionnaire was prepared for students in Grades 8–12 (corresponding to ages 14–18 years). These grades were chosen to ensure that participating students had reached puberty (which is commonly considered age 10–14 years in girls and 12–16 years in boys).

### Sampling and sample size

We used a non-probability convenience sampling technique. The questionnaire was disseminated to all schools in the seven Emirates via the administrative departments of education/Ministry of Education. The administrative departments directed the link of the electronic questionnaire to the administration of each school, who then disseminated the link to teachers and class masters during their online teaching. Using a 95% confidence level, 1% margin of error (alpha error), and response rate of 50% from a given population of approximately 130,000 secondary school students, we estimated that the minimum viable sample size for sufficiently powered analyzes was 894 students. The endpoint used to power the analysis assumed that ≥15% of students would consume CED daily (28).

### Study tools

The self-administered online questionnaire was prepared in both the Arabic and English languages and comprised structured, closed-ended questions. There were no open-ended or continuing questions, meaning the questionnaire was simple and quick to answer; it was estimated that the questionnaire would take around 10–15 min to complete based on a pilot test. The questionnaire covered six domains: first, sociodemographic (sex, nationality, Emirate, school type, grade, average grade, daily allowance, parental companionship, parental education, and occupational status) and anthropometric information (self-reported body weight and height); second, CED consumption (parental use of CED, frequency and amount of CED intake, source of information, health effects, reasons of consumption, and ingredients of CED); third, questions on diet (frequency of eating breakfast, fruits, vegetable, fast food, and energy-dense snack), and lifestyle (smoking, physical activity, smart device use) behaviors; fourth, self-reported physical health and frequency of reporting headache, stomach aches; fifth, self-reported mental health and frequency of reporting angry, nervous, anxious, or arguing, feeling lonely; and sixth, sleep quality parameters (night sleep duration, experiencing difficulty falling asleep).

### Exposures and outcomes

### Outcomes

The frequency of CED consumption was categorized as daily, three or more times/week, and less than three times/week. We then tested the associations between the frequency of CED consumption and sociodemographic factors, physical exercise habits, sleep habits, mental and physical health (self-reported), dietary habits, and parents’ education and employment status.

### Exposures

We evaluated the relationships between CED consumption (outcomes, dependent variables) and students’ health and well-being parameters (exposures, independent variables). Students’ nutritional status (as the prevalence of overweight and obesity), dietary behaviors servings of fruits and vegetables per day, eating/skipping breakfast, snacking on energy-dense snacks (such as cookies, ice cream, candies, crackers, chocolate bars, potato chips, soda), and eating fast foods (that energy-dense, easily prepared processed foods in the Westernized restaurants such as burgers, pizza, chicken or chips), lifestyle behaviors (physical activity level, smart device use, and smoking), and psychological characteristics were assessed as the main exposures. Sex, age (and corresponding school grade), nationality, place of residence (i.e., with whom the student lived), educational level, grade point average (GPA), and school type were the main moderators examined.

### Data analysis

Categorical variables were described using frequencies and percentages. Descriptive data, including mean, standard deviation, minimum, and maximum, were evaluated for weight-related variables (weight, height, and body mass index [BMI]). BMI was estimated based on students’ self-reported weight and height and then described as defined by the World Health Organization (WHO) growth chart z-scores for ages 5–19 years. These charts divide children (by z-scores) into five categories: less than −3 (severely thin), between −3 and − 2 (thin), between −2 and + 1 (normal), between +1 and + 2 (overweight), and more than +2 (obese). The growth chart cut-off points were applied to the whole population using Anthroplus Survey software. Parents’ and students’ CED consumption were described using frequencies and percentages. Students’ dietary habits, sleep habits, exercise habits, and physical and mental health (self-reported) were also described using frequencies and percentages. Smart device use was categorized as never, not every day, <2 h/day, >2 h/day. Sleep hours were grouped into two categories: ≤7 h, and ≥ 8 h. GPA was categorized as <80% or ≥ 80%. Physical activity was categorized as adequate (very good, good, and adequate) or inadequate (poor and very poor). Crosstabs and Pearson’s chi-square tests were used to evaluate the associations between the frequency of CED consumption (never, less than three times/week, three or more times/week, daily) and students’ sociodemographic data, sleep and physical activity data, self-reported physical and mental health, and parents’ data. The sub-sample of students who consumed CED was used for multiple-answer questions relating to these drinks (sorted by choice), and the frequencies and percentages for these items were obtained separately (100% did not cover the whole sample). Graphical illustrations were provided for sociodemographic, BMI, and multiple-answer data. All data were encoded and analyzed using IBM SPSS version 26.0. All significance levels were set at *p* < 0.05.

### Ethical considerations

Official permission to conduct this study was obtained from the Ministry of Education and ethical approval was obtained from the University of Sharjah Research Ethics Committee on September 24, 2021 (REC-21-06-18-01). This committee also approved the research protocol, informed consent, and research tools. Students were required to submit the online self-report questionnaire assessing their CED consumption, dietary and physical activity habits, and self-reported physical and mental health. Students completed the questionnaire in their regular class sections. Student and parent/guardian consent was necessary for participation in the study using an electronic consent form. Participation was voluntary; no monetary or non-monetary incentives were given and participants were informed that they could withdraw from this study at any time.

## Results

Our analysis included 4,648 students comprised of 3,021 males (65%) and 1,627 females (35%). More than half (54%) were non-Arab, followed by Emirati citizens (22.2%). The majority were from Sharjah (about 72%), followed by Ajman (13%) and Dubai (10%). The vast majority of participants were from private schools (88%). Nearly grades 8–10 contributed the same proportion of participants (22–23%), followed by grades 12 (18%) and 11 (14%). Around 40% of students had a GPA of ≥90%, followed by those who had 80–89% (32%). About 94% of students lived with their parents. The majority (73.2%) of students received a low daily allowance of <25 dirhams (about 7$) and 14.5% received 25–50 dirhams (6.75–13.5 $; Table 1). The majority of fathers (75%) and mothers (69%) had college- or university-level education. Most (91%) fathers were employed but only 34% of mothers were employed (Table 1). One-fifth (20%) of the students and 14% of parents consumed CED; only 2% of the student consumers used to drink CED daily (Table 2). In terms of dietary habits, more than two-thirds (about 68%) of students reported having breakfast every day, less than half (43%) had two or more servings of vegetables/day, and one-third (33%) had two or more servings of fruits/day. Almost 75% of students consumed one serving or less of fast food per week. Less than two-thirds (Around 65%) of students reported consuming energy-dense snacks twice or less/day. Almost all (98%) students were non-smokers. About half of the students were of normal weight, 23.8% were overweight, and 18.2% were obese (Table 2).

**Table 1:** Students’ sociodemographic and parental characteristics (*N* = 4,648).

Variable		*n* (%)
Sex	Male	3,035 (65.3)
	Female	1,613 (34.7)
Nationality	Emirati citizen	1,032 (22.2)
	GCC citizen	140 (3.0)
	Other Arab	964 (20.7)
	Non-Arab	2,512 (54.0)
Emirate	Sharjah	3,343 (71.9)
	Abu Dhabi	87 (1.9)
	Dubai	473 (10.2)
	Ajman	614 (13.2)
	Umm Al Quwain	29 (0.6)
	Ras Al Khaimah	25 (0.5)
	Fujairah	77 (1.7)
School type	Government/Public	560 (12.0)
	Private	4,088 (88.0)
School grade	Grade 8	999 (21.5)
	Grade 9	1,074 (23.1)
	Grade 10	1,079 (23.2)
	Grade 11	664 (14.3)
	Grade 12	832 (17.9)
Last grade point average	90% or more	1864 (40.1)
	80–89%	1,487 (32.0)
	70–79%	788 (17.0)
	60–69%	353 (7.6)
	50–59%	109 (2.3)
	50% or less	47 (1.0)
Parental companionship	Parents	4,356 (93.7)
	One parent (father or mother)	259 (5.6)
	Grandparents/relatives	22 (0.5)
	School care home/hostel	11 (0.2)
Daily allowance (AED/day) (0.27 USD/AED)	<25 (6.75USD)	3,404 (73.2)
	25–50 (6.75–13.5 USD)	675 (14.5)
	>50 (13.5 USD)	569 (12.2)
Father’s education level	Uneducated	41 (0.9)
	Primary	57 (1.2)
	Middle school	161 (3.5)
	High school	726 (15.6)
	College/university (including postgraduate)	3,499 (75.3)
	Do not know (not living with father)	164 (3.5)
Mother’s education level	Uneducated	79 (1.7)
	Primary	73 (1.6)
	Middle school	206 (4.4)
	High school	939 (20.2)
	College/university (including postgraduate)	3,215 (69.2)
	Do not know (not living with mother)	136 (2.9)
Father’s employment	Unemployed	249 (5.4)
	Employed	4,261 (91.7)
	Not applicable (do not know)	138 (3.0)
Mother’s employment	Unemployed	2,948 (63.4)
	Employed	1,578 (34.0)
	Not applicable (do not know)	122 (2.6)
BMI category	Severely thin	195 (4.2)
	Thin	251 (5.4)
	Normal	2,250 (48.4)
	Overweight	1,106(23.8)
	Obese	846 (18.2)

**Table 2:** Students’ CED consumption, and self-reported dietary and lifestyle habits (*N* = 4,648).

	*n* (%)
Do you consume CED?	
Yes	930 (20)
No	3,718 (80)
Frequency of CED consumption by users	930 (20)
<3 times/week	707 (15.2)
≥3 times/week	130 (2.8)
Daily	93 (2.0)
Drinking CED by parents	
Yes, one of the parents/guardians	525 (11.3)
Yes, both of them	181 (3.9)
No, none of the parents	3,663 (78.8)
Do not know	279 (6.0)
How often do you have breakfast?	
Daily	3,137 (67.5)
Not daily	1,511 (32.5)
How many servings of vegetables do you have per day (one serving equals about one medium cucumber, tomato, carrot, or half a cup of cooked vegetables)?	
1 serving or less	2,649 (57.0)
2 servings or more	1,999 (43.0)
How many servings of fruits or fresh fruit juices do you have per day (one serving of fruit is about the size of a medium apple or a small banana, and one serving of fruit juice is about half a cup)?	
1 serving or less	3,123 (67.2)
2 servings or more	1,525 (32.8)
How many times per week do you consume fast food?*	
1 time or less	3,472 (74.7)
2 times or more	1,176 (25.3)
How many times do you have energy-dense snacks per day (e.g., chips, ice cream, candy, cookies)?*	
Less than 2 times	2,998 (64.5)
2 or more times	1,650 (35.5)
Are you a smoker (cigarettes, hookah/shisha, smoking pipe (*midwakh*), e-cigarette/vape)?	
No	4,532 (97.5)
Yes	116 (2.5)

*Energy-dense snacks such as cookies, ice cream, candies, crackers, chocolate bars, potato chips, and soda. Fast foods are energy-dense, easily prepared processed foods in Westernized restaurants such as burgers, pizza, chicken, or chips.

**Figure 1:**
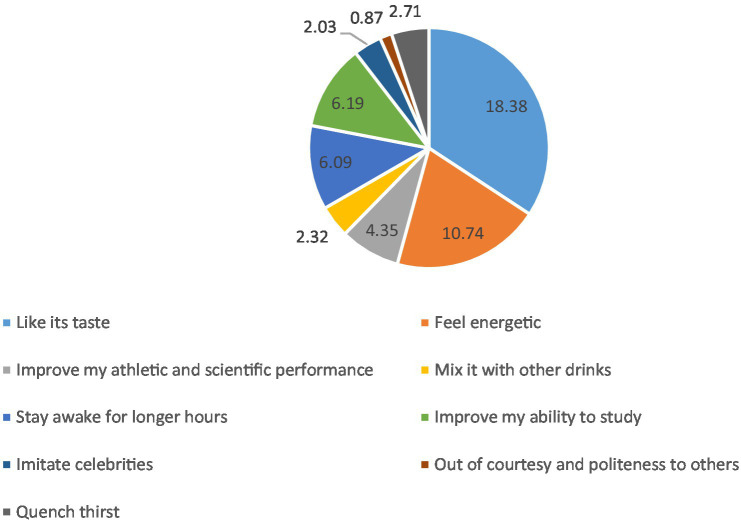
Reasons for consuming CED by schoolchildren (%) (*N* = 1,034; multiple answers).

**Figure 2:**
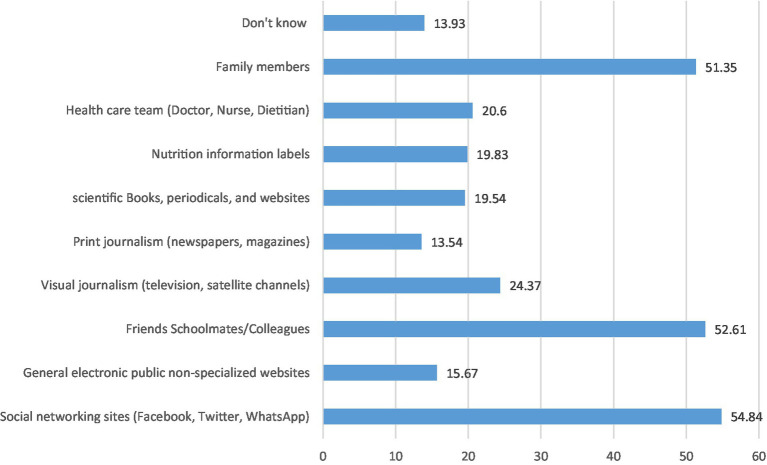
Sources of information about CED as per the perspectives of CED consumers (*N* = 1,034; multiple answers).

**Figure 3:**
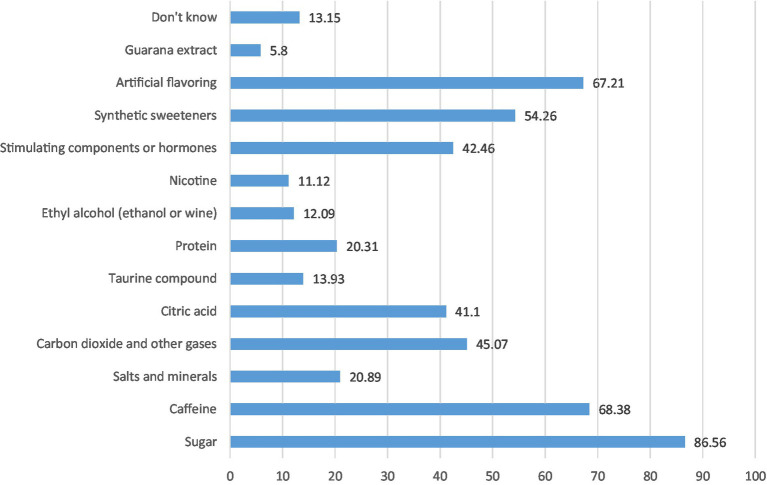
Ingredients of CED as reported by the CED consumers (*N* = 1,034; multiple answers) (%). *The ingredients listed reflect the perspectives of CED consumers and do not imply that they are present in CED.

**Figure 4:**
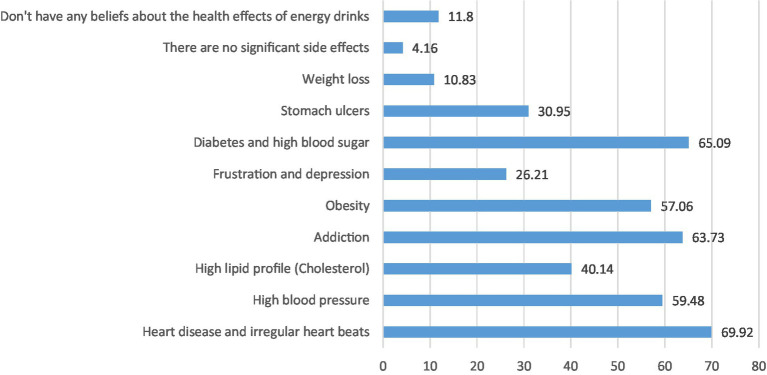
Opinions of CED consumers (%) about the long-term negative health effects associated with CED (*N* = 1,034; multiple answers). *Changes listed reflect the perspectives of CED consumers and do not imply that they are scientifically proven effects.

Reasons for consuming CED were their taste (18%), feeling energetic (11%), improving the ability to study (6%), and staying awake longer (6%) (Figure 1). Sources of information about CED among participants who consumed CED were social networking sites (around 55%), friends and schoolmates/peers (53%), and family members (51%) (Figure 2). Students reported some misconceptions about the ingredients of CED, although most (87%) believed that they contained sugar. More than two-thirds were aware of the CED content of caffeine (68%) and artificial flavoring (67%), fewer reported that CED may contain synthetic or artificial sweeteners (54%), carbon dioxide, and other gasses (45%), stimulating components or hormones (43%) and citric acid (41%) (Figure 3). Evaluation of students’ awareness about the long-term negative effects of the consumption of CED on their health showed commonly mentioned issues were heart disease and irregular heartbeat (70%), diabetes and high blood sugar (65%), addiction (64%), high blood pressure (59.5%), obesity (57%), and high blood lipids (40%) (Figure 4).

About two-thirds of the participating schoolchildren used smart devices <2 h per day (67%), exercised less than three times/week (62%), suffered from frequent headaches (64%), and suffered from frequent stomach aches (74%). About two-thirds (65%) of students described their physical activity as good-very good, about 56% described their mental health as good-very good, only 16% reported ≥8 h of sleep per night, and about 35% had difficulty falling asleep. About half-to-half reported the presence-absence of being frequently angry, nervous, anxious, or arguing (Table 3).

**Table 3:** Daily behaviors and self-reported physical and mental conditions of the study participants (*N* = 4,648).

Practices and symptoms	*n* (%)
Use tablets/smart devices (excluding remote study hours) every day	
Never	111 (2.4)
Not every day	380 (8.2)
>2 h/day	1,066 (22.9)
<2 h/day	3,091 (66.5)
Times do exercise per week	
<3 times/week	2,883 (62.0)
≥3 times/week	1765 (38.0)
Suffer from frequent headaches	
Yes	2,951 (63.5)
No	1,696 (36.5)
Suffer from frequent stomach aches	
Yes	3,427 (73.7)
No	1,221 (26.2)
Self-reported physical health	
Very good	895 (19.3)
Good	2,108 (45.4)
Adequate	1,243 (26.7)
Poor	331 (7.1)
Very poor	71 (1.5)
Self-reported mental health	
Very good	971 (20.9)
Good	1,610 (34.6)
Adequate	1,124 (24.2)
Poor	559 (12.0)
Very poor	382 (8.2)
Hours usually sleep on a school night	
<6	1,411 (30.4)
6–7	2,473 (53.2)
≥8	764 (16.4)
Have experienced any difficulty falling asleep	
Yes	1,608 (34.6)
No	3,040 (65.4)
Frequently angry, nervous, anxious, or arguing	
Yes	2,295 (49.4)
No	2,351 (50.6)
Often feel lonely	
Yes	1864 (40.1)
No	2,784 (59.9)

The relationship between sociodemographic variables and CED consumption is presented in Table 4. Females reported a significantly lower intake of CED (*p* = 0.001) than males. There was a significant difference in CED consumption by nationality (*p* = 0.001), with non-Arab students reporting significantly lower consumption than all other nationalities (*p* = 0.001). There was a significant association between CED consumption and school grade (*p* = 0.001), with consumption significantly higher in higher grades (i.e., Grade 12 vs. Grades 8 and 9; *p* = 0.001).

**Table 4:** Association between CED consumption and students’ sociodemographic characteristics (*N* = 4,648).

Variable		Frequency of CED consumption (% of the population)				*p*-value
		Daily (93, 2%)	≥3 times/week (130, 2.8%)	<3 times/week (707, 15.2%)	Never (3,718, 80%)	
Sex	Females	1.0	1.4	9.0	53.9	0.001*
	Males	1.0	1.4	6.2	26.1	
Nationality^#^	Emirati citizen^a^	1.0	1.2	5.2	14.8	0.001*
	GCC citizen^b^	0.01	0.2	0.4	2.4	
	Other Arab^b^	0.6	0.9	3.6	15.7	
	Non-Arab^c^	0.5	0.5	0.6	47.1	
School grade^#^	Grade 8^a^	0.4	0.5	2.3	18.3	0.001*
	Grade 9^a^	0.3	0.5	3.1	19.2	
	Grade 10^b^	0.5	0.6	3.7	18.4	
	Grade 11^bc^	0.4	0.5	2.6	10.9	
	Grade 12^c^	0.5	0.8	3.5	13.1	
GPA^#^	≥90%^c^	0.9	1.2	5.5	32.6	0.001*
	80–89%^c^	0.6	1.0	5.3	25.1	
	70–79%^bc^	0.3	0.4	2.8	13.5	
	60–69%^bc^	0.1	0.1	1.1	6.2	
	50–59%^bc^	0.0	0.0	0.4	1.9	
	<50%^a^	0.1	0.1	0.2	0.6	
School type	Private	1.6	2.4	13.0	71.0	0.001*
	Governmental/public	0.4	0.4	2.2	9.0	
Parental companionship^#^	Parents^a^	1.7	2.4	13.8	75.8	0.001*
	One parent^b^	0.2	0.3	1.3	3.7	
	Grandparents/ relatives^a^	0.0	0.0	0.1	0.3	
	School care home/hostel^a^	0.1	0.0	0.0	0.1	
Daily allowance (AED/day)^#^ (0.27 USD/AED)	<25 (6.75USD)^a^	0.7	1.3	9.1	62.1	0.001*
	25–50 (6.75–13.5 USD)^b^	0.5	0.5	2.6	10.9	
	>50 (13.5 USD)^c^	0.9	1.0	3.5	6.9	
BMI (percentiles)	Severely thin	0.1	0.1	1.4	8.0	0.337
	Thin	0.2	0.3	1.3	7.5	
	Normal	0.9	1.5	8.0	40.8	
	Overweight	0.5	0.4	2.7	13.5	
	Obese	0.2	0.3	2.1	10.2	

*P-value obtained using crosstabs and chi-square test. ^#^Different letters in the same column for each variable indicate significance between groups using ANOVA–*Post Hoc* analysis, *Significant at *P* < 0.05. BMI, body mass index; GPA, grade point average.

As shown in Table 4, Students with a GPA of ≤50% reported significantly higher consumption of CED compared with those with higher GPA levels (*p* = 0.001). Schoolchildren in governmental/public schools students reported significantly higher consumption of CED compared with their counterparts in private schools (*p* = 0.001). Students who lived with both parents had significantly (*p* = 0.001) lower consumption of CED compared with all types of companionship. Those who had a low daily allowance (≤ 25 Emirati dirhams AED, about 7 $ or less) had the lowest CED consumption, while students with a high daily allowance (>50 AED, about 14 $ or more) had the highest consumption of CED (*p* = 0.001; Table 5).

**Table 5:** Association between CED consumption and physical exercise, sleep, and physical and mental health (%).

Variable		Frequency of CED consumption				*P*-value
		Daily (93, 2%)	≥3 times/week (130, 2.8%)	<3 times/week (707, 15.2%)	Never (3,718, 80%)	
Using smart devices^#^	Never ^ab^	2.7	2.7	9.9	84.7	0.001*
	Not every day ^ab^	1.3	2.6	15.0	81.1	
	<2 h/ day ^a^	0.6	1.9	10.6	87.0	
	>2 h/ day ^b^	2.6	3.1	17.0	77.3	
Practicing exercise/weekly	<3 times/week	1.8	2.3	13.9	82.0	0.001*
	≥3 times/week	2.4	3.6	17.3	76.7	
Headaches	Yes	3.3	4.3	18.3	74.1	0.001*
	No	1.3	1.9	13.5	83.4	
Stomach aches	Yes	4.2	19.2	4.8	71.8	0.001*
	No	1.2	13.8	2.1	82.9	
Describing physical health^#^	Very good ^a^	2.9	3.5	16.0	77.7	0.024*
	Good ^b^	1.4	2.4	14.3	81.9	
	Adequate ^a^	1.4	2.7	15.7	80.1	
	Poor ^ac^	3.0	3.6	17.5	75.8	
	Very poor ^ac^	13.0	4.3	15.9	66.7	
Describing mental health^#^	Very good ^ac^	2.8	2.6	12.8	81.9	0.001*
	Good ^a^	1.1	1.9	13.0	84.0	
	Adequate ^c^	1.2	2.4	16.2	80.2	
	Poor ^b^	2.0	3.9	18.4	75.7	
	Very poor ^b^	6.3	6.5	23.3	63.9	
Sleep hours on school nights^#^	<6 h ^a^	3.1	2.1	12.8	81.9	0.001*
	6–7 h ^a^	1.1	1.7	13.8	83.4	
	≥8 h ^b^	3.0	5.0	19.0	72.9	
Difficulty falling asleep	Yes	3.0	4.9	20.8	71.2	0.001*
	No	1.4	1.7	12.3	84.6	
Feeling angry, nervous, anxious, arguing	Yes	2.7	3.7	18.3	75.3	0.001*
	No	1.4	1.9	12.3	84.5	
Feeling lonely	Yes	2.8	3.9	17.2	76.1	0.001*
	No	1.4	2.0	13.9	82.6	

*P-value obtained using Crosstabs and chi-square test. ^#^Different letters in the same column for each variable indicate significance between groups using ANOVA–*Post Hoc* analysis, Scheffe test.*Significant at *P* < 0.05. Percentages are calculated per row.

A significant positive association was found between CED consumption and students’ use of smart devices (*p* = 0.001). Among those who never consumed CED, significantly more students had lower time duration for using smart devices (<2 h/day vs. >2 h/day, 87% vs. 77%; *p* = 0.001). Students who exercised more than three times/week had significantly higher CED consumption than those who exercised three or fewer times/week (*p* = 0.001). Students who reported headaches consumed significantly more CED than those without headaches (*p* = 0.001). The same trend was reported for stomach aches, students who consumed significantly more CED witnessed more stomach aches than those who did not consume CED (*p* = 0.001). Furthermore, those who reported “good physical health” had significantly less CED consumption than the other groups (*p* = 0.024). The same trend was reported or self-reported mental health, those who reported “good-very good mental health” had significantly less CED consumption than the other groups with higher CED consumption (*p* = 0.001).

Unexpectedly, there was a direct and significant association between CED consumption rate and the number of sleep hours per night on school days (*p* = 0.001). Students who slept <6 h/night and 6–7 h/night reported significantly lower CED consumption than those who slept ≥8 h/night (*p* < 0.05). Nonetheless, those who reported less CED consumption had reported significantly less difficulty falling asleep (*p* = 0.001). Moreover, those who reported feeling angry, nervous, anxious, or arguing reported significantly (*p* = 0.001) more CED consumption than those who did not. Along the same line, students who reported feeling lonely had significantly (*p* = 0.001) more CED consumption than those who did not feel lonely.

Students with uneducated fathers reported significantly more CED consumption than those children with all other levels of fathers’ education (*p* = 0.001). Similarly, students with mothers who had college/university education consumed significantly (*p* = 0.001) fewer CED than those with mothers who reported less educational level (Table 6). Students with employed fathers reported significantly (*p* = 0.001) fewer CED consumption than those with unemployed fathers. On the contrary, students with employed mothers reported significantly (*p* = 0.001) more CED consumption than those with unemployed mothers. Students who used to eat breakfast daily reported significantly (*p* = 0.001) fewer CED consumption than those who did not use to eat this meal. Students who consumed one serving or less of vegetables/day were significantly (*p* = 0.001) more likely to consume CED than those who consumed two or more servings of vegetables/day. However, no such association was found for fruit consumption. Students who used to frequently consume fast foods (energy-dense, easily prepared processed foods in Westernized restaurants) reported significantly (*p* = 0.001) more CED consumption than those who reported less frequent fast-food consumption. Moreover, students who used to frequently consume energy-dense snacks reported significantly (*p* = 0.001) more CED consumption than those with less frequent snacking. Though very small in number, smokers reported significantly (*p* = 0.001) more CED consumption than their non-smoker counterparts (Table 6).

**Table 6:** Association between CED consumption and parental factors.

Variable		Frequency of CED consumption				*P*-value
		Daily (93, 2%)	≥3 times/week (130, 2.8%)	<3 times/week (707, 15.2%)	Never (3,718, 80%)	
Fathers’ educational level^#^	Uneducated^a^	9.8	12.2	36.6	41.5	0.001*
	Primary^bcd^	1.8	1.8	17.5	78.9	
	Middle school^bcd^	3.7	5.0	18.6	72.7	
	High school^bd^	3.2	3.7	18.2	74.9	
	College/university^c^	1.5	2.2	13.9	82.5	
	Do not know^d^	4.3	7.6	21.3	66.5	
Mothers’ educational level^#^	Uneducated^abc^	1.5	5.1	22.8	67.1	0.001*
	Primary^abc^	0.0	9.6	11.0	79.5	
	Middle school^b^	1.9	1.9	26.2	69.9	
	High school^b^	2.1	3.5	17.0	77.3	
	College/university^c^	1.7	2.2	13.8	82.3	
	Do not know^b^	7.4	7.4	18.4	66.9	
Fathers’ employment^#^	Unemployed^a^	4.0	6.4	17.3	72.3	0.001*
	Employed^b^	1.8	2.4	15.1	80.8	
	Do not know^a^	5.1	8.7	16.7	69.6	
Mothers’ employment^#^	Unemployed^a^	1.8	2.3	14.5	81.5	0.001*
	Employed^b^	2.0	3.4	15.9	78.6	
	Do not know^c^	7.4	6.6	25.4	60.7	
Consuming breakfast	Daily	1.1	1.6	12.5	84.9	0.001*
	Not daily	4.0	5.4	21.0	69.7	
Vegetables serving/day	≤1 serving	2.7	3.2	15.8	78.3	0.001*
	≥2 servings	1.1	2.3	14.5	82.2	
Fruits serving/day	≤1 serving	2.0	2.8	14.2	80.9	0.053
	≥2 servings	2.0	2.7	17.3	78.0	
Fast foods/week**	≤1 time	1.1	1.5	12.6	84.8	0.001*
	≥2 times	4.6	6.7	23.1	65.6	
Energy-dense snacks/day**	<2 times	1.0	1.9	11.8	85.3	0.001*
	≥2 times	3.9	4.4	21.4	70.3	
Smoking	Yes	14.7	16.4	30.2	38.8	0.001*
	No	1.7	2.5	14.9	81.0	

The *p*-value was obtained using Crosstabs and the chi-square test. ^#^Different letters in the same column for each variable indicate significance between groups using ANOVA–post hoc analysis, Scheffe test. *Significant at *p* < 0.05. Percentages are calculated per row. College/university educational level includes postgraduate. “Do not know” at the educational level indicates not living with a father/mother. **Energy-dense snacks such as cookies, ice cream, candies, crackers, chocolate bars, potato chips, and soda. Fast foods are energy-dense, easily prepared processed foods in Westernized restaurants such as burgers, pizza, chicken, or chips.

## Discussion

This survey-based research explored for the first time, at the national scale, the prevalence of CED consumption and their associations with sociodemographic, nutritional, dietary, lifestyle, and sleep characteristics of the schoolchildren in the UAE. We found that 20% of students consumed CED. Students learned about CED from different unreliable sources, such as social media, friends, schoolmates, and family members, and the majority were aware that consumption of CED was associated with adverse health consequences. We found significant associations between students’ CED consumption and various sociodemographic and health indicators, including nationality, place of residence, sex, type of school, daily allowance, GPA, physical and mental health, lifestyle and dietary habits, use of smart devices, sleep characteristics, and timing, perceived mental and physical health, and parental education level and employment status.

Our findings on the prevalence of CED consumption among schoolchildren are generally consistent with previous findings from the UAE (27%) (30) and close to one study from Australia (24%) (32). However, the reported prevalence is much lower than what was found in other parts of the globe, where the prevalence ranged from 28.0–66% (6, 16–27). Considering the similar economic and socioeconomic backgrounds in the GCC countries, the prevalence of CED consumption in the UAE is much lower than the reported high prevalence in Saudi Arabia (45–60%) among middle and secondary schoolchildren (28, 29). This relatively lower prevalence of CED consumption among school children in the UAE could be explained by the legal restriction on selling CED to children under 16 years old, which is announced and declared in supermarkets and large groceries and has been banned in school canteens (33, 34). Further, the increased awareness of parents and families about the adverse consequences of CED on children’s health may explain this notion (35).

The reported association between the male sex and CED consumption has been examined by several studies (25, 36, 37). Consistent with studies on school and college students, male students were much more likely to report CED consumption than females (11, 32, 37–40). This clear tendency by males could be explained by the masculinity ideology that characterizes young adolescent males (41). Such ideology states that male adolescents tend to have more stereotypically masculine beliefs driven by the male sex hormones (42), making them more likely to believe that CED work better, which, in turn, leads to drinking more of these formulated drinks (43).

The reported obvious differences between Arab and non-Arabs concerning the consumption of CED could be ascribed to the differences in the health education, knowledge, and family effect on children’s food selection and decisions, which are found to be largely affected by the ethnicity and racial background (44, 45). The reported higher CED consumption by higher grades is consistent with previous studies (18, 46) and could be explained by the role of sex hormones and an increased feeling of power and dependence associated with the increased age of students, especially male students (47).

The role of caffeine derived from CED in stimulating the central nervous system and increasing alertness leads cognitive function to the assumption that frequent CED consumption is expected to promote academic achievement presented in higher cumulative grade point average (GPA) (48). This proposed effect has been investigated by Champlin et al. (49) among college students. Consistent with our results on schoolchildren, Champlin and colleagues found that drinking CED was associated with a lower GPA even after controlling for potential confounding variables. Further in Pakistan, no relationship was found between the GPA and CED consumption among university students (50).

Daily allowance, which reflects the purchasing power and socioeconomic level of the school children, has a clear direct relationship with CED consumption. This was evident in our results and also corroborated by relevant research on university students in Beirut, Lebanon (51) and among adolescents in Australia (32) where the economic levels of the students positively determined the extent and type of CED consumption. However, this finding contradicts what was reported in Norway, where a larger proportion of high CED consumers was associated with adolescents with lower socioeconomic status (52). Albeit students in private schools reported higher purchasing power to afford the CED, they reported a lower prevalence of CED consumption than their counterparts in the public schools, a matter that could be interpreted by the level of knowledge and awareness about health sequelae of CED, rather than the economic pr purchasing power that determines the tendency to purchase and consume CED.

Family relationship presented in parental companionship is an integral component for healthy children’s growth and development and ensuring their mental and social well-being (53). This is clearly reflected in our results, where the schoolchildren with parental companionship exhibited a significantly lower prevalence of CED consumption than other groups of companionship.

The direct relationship between CED consumption with smart device use and screen time is consistent with and mirrors the results of several previous studies in different countries (30, 37, 52, 54, 55). In the United States, a large-scale national study on 32,418 schoolchildren from 252 to 263 schools (grades 8–10) revealed that each hour per day talking on a cellphone was associated with an increased risk of exceeding WHO caffeine intakes by 18% (OR = 1.15–1.21; *p* < 0.001), and only video game use was weakly linked to caffeine intake, with the latter comes mostly from CED (56). This notion was further confirmed by Larson et al. on 2,793 American adolescents in grades 6–12 (54).

The relationship between physical activity and CED consumption has been repeatedly examined among adolescents and adults with contradicting results reached (8, 30, 37, 52, 54, 55, 57). Consistent with our results, Puupponen et al. (8) found no association between frequent CED consumption and low physical activity and confirmed that frequent CED consumers showed a relatively higher likelihood of reporting high physical activity than non-consumers. Our and Puupponen’s findings disagree with the reported negative relationship between CED consumption and PA levels among Norwegian adolescents. The tendency of physically active students to consume CED comes from the fact that CED are purported and advertised to increase mental concentration as well as physical performance and help in achieving goals and winning competitions (58).

Headaches and stomach aches are known as common side effects of the excessive intake of caffeine (59, 60). Hence, the coexistence of more reported headaches and stomach aches among those with more frequent consumption of CED is expected, and comes in line with the existing literature linking CED with such adverse symptoms (61, 62). Reporting headaches and stomach aches are confirmed by relevant and recent reports among Icelandic adolescents who were exposed to excessive caffeine (62). Such unpleasant effects could be explained by the fact that excessive caffeine intake induces a pro-nociceptive state of cortical hyperexcitability (59), which in turn triggers such symptoms.

The poor self-reported physical and mental health among CED users reveals a plausible association between worsened physical and mental health with the consumption of CED among schoolchildren. Such a finding is consistent with what Halldorsson and colleagues found in their study on Icelandic adolescents (62). Further, they reported emotional and behavioral problems among adolescents who were exposed to CED (63). This is consistent with the several negative health indicators reported by Puupponen and colleagues (8). In the latter, even infrequent CED consumption was associated with adverse health indicators, while frequent CED consumers were more likely to report skipping breakfast, inadequate tooth brushing, current problematic social media use, smoking, snus, and cannabis use, feelings of insufficient sleep and reporting short sleep, low self-rated health, and multiple health complaints (8). All the aforementioned negative health indicators are consistent with the self-reported poor physical and mental health found among the CED consumers in the current work.

Though unexpected results indicate a positive relationship between CED consumption and sleep duration (i.e., sleep night hours), the difficulty in falling asleep is indicative of the adverse effect of CED on sleep quality. Sleep quality includes, but is not limited to, sleep hours and other six components as indicated in the Pittsburgh Sleep Quality Index (PSQI) (64). Having prolonged time to falling asleep could be explained by both the stimulatory effect of caffeine (the main ingredient of CED) and the plausible excessive exposure to blue light from the prolonged use of smart devices, especially if used at night, by the CED consumers. Both caffeine and the blue light radiated from smart devices’ screens have stimulatory effects on the CNS and may be involved in delaying sleep onset. According to the Sleep Foundation, the use of smart devices that emit artificial blue light in the evening and at night disrupts the natural sleep–wake cycle by preventing the brain from producing melatonin prior to bedtime (65). Our current finding on self-reported sleep duration contradicts several research works that indicated that CED consumption was associated with reduced sleep duration and sleep quality as well (5, 30, 32, 40, 54, 57, 62, 66, 67). Such discrepancy could be ascribed to the lack of utilization of validated subjective sleep quality questionnaires such as PSQI in the current work. Interestingly, the lack of sleep and skipped breakfast were the affectors that mediate emotional and behavioral problems that characterize CED consumers among 8,405 adolescents from 11 to 15 years old (63).

The reported association between unhealthy dietary behaviors such as breakfast skipping and a lower intake of fruits and vegetables with CED consumption mirrors the findings of other relevant reports (15, 54, 68). However, Nuss et al. (32) did not find such a negative relationship between fruit and vegetable consumption. Regarding frequent snacking and consumption of energy-dense fast foods, the observed association between CED consumption and these dietary patterns is also consistent with previous works (30, 32, 54, 69).

The obvious and noticeable coexistence of unhealthy dietary behavior with other unhealthy and risky lifestyle behaviors such as smoking and long screen time, could be explained by the low health awareness that characterizes those children and their families, a matter that needs further investigation to find out the clustering of these behaviors in the same group of participants.

Although the proportion of smoking school students was very low, CED consumption was predominant among those smoking children. This relationship between smoking and CED consumption is repeatedly documented in several reports (6, 8, 9, 46, 54) and could be ascribed to the clustering of unhealthy behaviors alongside with lack of awareness about the unhealthy dietary and lifestyle behaviors and their adverse health sequelae. As for schoolchildren and adolescents, these behaviors and the lack of knowledge and awareness are mostly a reflection of the family’s educational and awareness levels and the effect of peers (45, 70). In their extensive systematic review, Marinoni et al. (70) found that schoolchildren and adolescents with low parental monitoring, with lower grades, or those exposed to bad influences from peers are more likely to consume CED. They concluded that the consumption of CED by schoolchildren and adolescents lies in the potential interplay between influences by family members and peers, personality traits, and school performance.

In the current work, the increased reporting of poor self-reported mental health signs such as feeling angry, nervous, anxious, and lonely along with emotional and behavioral problems are evident for adolescents with CED consumption as reported in previous works (13, 17–19, 63, 68).

The overall adverse health effects of CED are exacerbated by the industry’s aggressive marketing directed to adolescents, and the absence of activated regulatory and parental supervision. To combat this growing public health concern, policymakers should consider creating a discrete regulatory category for CED, establishing an evidence-based upper limit on caffeine, insisting on the application of restricted CED sales for schoolchildren and adolescents, and enforcing regulating existing CED marketing strategies, particularly among schoolchildren and adolescents (12).

### Strengths and limitations

This was the first study to evaluate the prevalence of CED consumption among school students and examine its associations with various dietary and lifestyle- and health-related factors in the UAE. This study was conducted on a large scale, and participants were relatively evenly distributed across different school grades (Grades 8–12, corresponding to 14–18 years old). Therefore, our findings offer good evidence of the need to address issues around the consumption of CED among schoolchildren in the UAE. Moreover, our database serves as a valuable resource to further examine these parameters in the context of diverse sociodemographic variables, given the rich presence of different ethnicities in the UAE. However, the observational nature of this study means we cannot infer causality. Further, relying on self-reported CED consumption and other outcomes, the study can be susceptible to both social desirability bias and recall bias (52). Moreover, the associations between CED consumption and self-reported physical and mental health variables may be affected by the fact that caffeine, the principal active component of CED, is present in different foods and drinks along with other nutrients that may influence physical and mental health. In addition, only students who were in school on the day of the survey responded to the questionnaire. Therefore, adolescents who were absent or had pulled out were excluded from the study, which may affected the findings. Further, the BMI was calculated based on self-reported weight and height. The absence of comprehensive assessment tools, such as PSQI, and other validated measures for mental health and eating behaviors, lessens the robustness of the current findings and necessitates additional research to enhance their power. Lastly, the limited and unrepresentative sample size for the UAE schoolchildren makes the generalizability of the current results unattainable.

### Policy implications

Our findings have broad policy implications for improving the provision of health services and health behaviors among UAE schoolchildren and adolescents. Our findings could also serve as a useful guideline for implementing government initiatives to improve school physical and mental health and nutrition, reduce childhood obesity, and prevent children from smoking. Given the potential health risks associated with CED, the UAE government has implemented policies to regulate CED consumption. For example, CED are only sold to customers over the age of 16 years, warning labels must be displayed on the packages of these drinks, and there is a tax on CED to discourage consumption. Overall, the UAE’s CED-related policies are intended to protect the health of young people by limiting their access to these potentially harmful products. Although the effectiveness of these policies is still being evaluated, they represent an important step toward promoting the health and well-being of UAE children and adolescents. Several interventions may be useful, such as specific rules about consuming caffeine and soft drinks in schools, the exclusion of vending machines selling soft drinks from school premises, providing safe drinking water, school-based health campaigns, the imposition of high taxes on caffeine and CED, and limiting their promotion and advertising in social media. In addition, the UAE launched a Family Protection Policy, which seeks to strengthen social ties in UAE families and communities (71). It is also important to develop innovative child/adolescent-friendly initiatives and secure their participation by including their views when considering overall protection and their best interests (72).

## Conclusion

There is a relatively low prevalence of CED consumption among schoolchildren and adolescents in the UAE. Still, those who consumed CED have reported unhealthy dietary (skipping breakfast, frequent snacking and eating fast foods, low fruit, and vegetable intake) and lifestyle behaviors (long screen time, poor sleep quality component), accompanied by poor self-reported mental and physical health. Variable sociodemographic variables, such as nationality, parental companionship, school type, sex, education level, daily allowance, and parents’ employment and educational levels interplay with CED consumption among schoolchildren in the UAE.

## Data availability statement

The raw data supporting the conclusions of this article will be made available by the authors, without undue reservation.

## Ethics statement

The studies involving humans were approved by ethical approval was obtained from the University of Sharjah Research Ethics Committee on September 24, 2021 (REC-21-06-18-01). The studies were conducted in accordance with the local legislation and institutional requirements. Written informed consent for participation in this study was provided by the participants’ legal guardians/next of kin.

## Author contributions

MF: Conceptualization, Investigation, Methodology, Project administration, Supervision, Validation, Writing – original draft, Writing – review & editing. FA: Writing – review & editing, Supervision. MI: Writing – original draft. DA: Formal analysis. ES: Investigation, Writing – review & editing. ET: Investigation, Writing – review & editing. MA-K: Investigation, Writing – review & editing. SA-Q: Investigation, Writing – review & editing. FZ: Writing – review & editing. HH: Writing – review & editing. MH: Writing – review & editing. TO: Writing – review & editing. HR: Writing – review & editing. LC: Writing – review & editing. FN: Writing – review & editing. FB: Writing – review & editing. RO: Writing – review & editing.

## References

[1] Khouja C Kneale D Brunton G Raine G Stansfield C Sowden A. Consumption and effects of caffeinated energy drinks in young people: an overview of systematic reviews and secondary analysis of UK data to inform policy. BMJ Open. (2022) 12:e047746. doi: 10.1136/bmjopen-2020-047746, PMID: 35131813 PMC8830236

[2] Li P Haas NA Dalla-Pozza R Jakob A Oberhoffer FS Mandilaras G. Energy drinks and adverse health events in children and adolescents: a literature review. Nutrients. (2023) 15:2537. doi: 10.3390/nu15112537, PMID: 37299498 PMC10255861

[3] Oddy WH O’Sullivan TA. Energy drinks for children and adolescents. BMJ. (2009) 339:b5268. doi: 10.1136/bmj.b5268 20008969

[4] Alhyas L El Kashef A AlGhaferi H. Energy drinks in the Gulf cooperation council states: a review. JRSM Open. (2016) 7:2054270415593717. doi: 10.1177/2054270415593717, PMID: 26770815 PMC4710126

[5] Watson EJ Banks S Coates AM Kohler MJ. The relationship between caffeine, sleep, and behavior in children. J Clin Sleep Med. (2017) 13:533–43. doi: 10.5664/jcsm.6536, PMID: 28162144 PMC5359329

[6] Gallimberti L Buja A Chindamo S Vinelli A Lazzarin G Terraneo A. Energy drink consumption in children and early adolescents. Eur J Pediatr. (2013) 172:1335–40. doi: 10.1007/s00431-013-2036-1, PMID: 23708215

[7] Glade MJ. Caffeine–not just a stimulant. Nutrition. (2010) 26:932–8. doi: 10.1016/j.nut.2010.08.004, PMID: 20888549

[8] Puupponen M Tynjälä J Välimaa R Paakkari L. Associations between adolescents’ energy drink consumption frequency and several negative health indicators. BMC Public Health. (2023) 23:258. doi: 10.1186/s12889-023-15055-6, PMID: 36747163 PMC9903583

[9] Visram S Cheetham M Riby DM Crossley SJ Lake AA. Consumption of energy drinks by children and young people: a rapid review examining evidence of physical effects and consumer attitudes. BMJ Open. (2016) 6:e010380. doi: 10.1136/bmjopen-2015-010380, PMID: 27855083 PMC5073652

[10] Cadoni C Peana AT. Energy drinks at adolescence: awareness or unawareness? Front Behav Neurosci. (2023) 17:1080963. doi: 10.3389/fnbeh.2023.1080963, PMID: 36891321 PMC9986288

[11] Arria AM Bugbee BA Caldeira KM Vincent KB. Evidence and knowledge gaps for the association between energy drink use and high-risk behaviors among adolescents and young adults. Nutr Rev. (2014) 72:87–97. doi: 10.1111/nure.12129, PMID: 25293548 PMC4196711

[12] Al-Shaar L Vercammen K Lu C Richardson S Tamez M Mattei J. Health effects and public health concerns of energy drink consumption in the United States: a Mini-review. Front Public Health. (2017) 5:225. doi: 10.3389/fpubh.2017.00225 28913331 PMC5583516

[13] Holubcikova J Kolarcik P Madarasova Geckova A Reijneveld SA van Dijk JP. Regular energy drink consumption is associated with the risk of health and behavioural problems in adolescents. Eur J Pediatr. (2017) 176:599–605. doi: 10.1007/s00431-017-2881-4, PMID: 28229268

[14] Gambon DL Brand HS Boutkabout C Levie D Veerman ECI. Patterns in consumption of potentially erosive beverages among adolescent school children in the Netherlands. Int Dent J. (2011) 61:247–51. doi: 10.1111/j.1875-595X.2011.00067.x, PMID: 21995371 PMC9374831

[15] Puupponen M Tynjälä J Tolvanen A Välimaa R Paakkari L. Energy drink consumption among Finnish adolescents: prevalence, associated background factors, individual resources, and family factors. Int J Public Health. (2021) 66:620268. doi: 10.3389/ijph.2021.620268 34744582 PMC8565280

[16] Girán J Girán KA Ormándlaky D Pozsgai É Kiss I Kollányi Z. Determinants of pupils' energy drink consumption – findings from a Hungarian primary school. Heliyon. (2023) 9:e15954. doi: 10.1016/j.heliyon.2023.e15954, PMID: 37206032 PMC10189414

[17] Tóth Á Soós R Szovák E Najbauer N M Tényi D Csábí G. Energy drink consumption, depression, and Salutogenic sense of coherence among adolescents and young adults. Int J Environ Res Public Health. (2020) 17:1290. doi: 10.3390/ijerph17041290, PMID: 32079347 PMC7068601

[18] Masengo L Sampasa-Kanyinga H Chaput J-P Hamilton HA Colman I. Energy drink consumption, psychological distress, and suicidality among middle and high school students. J Affect Disord. (2020) 268:102–8. doi: 10.1016/j.jad.2020.03.004, PMID: 32157999

[19] Utter J Denny S Teevale T Sheridan J. Energy drink consumption among New Zealand adolescents: associations with mental health, health risk behaviours and body size. J Paediatr Child Health. (2018) 54:279–83. doi: 10.1111/jpc.13708, PMID: 28905482

[20] Frayon S Wattelez G Cherrier S Cavaloc Y Lerrant Y Galy O. Energy drink consumption in a pluri-ethnic population of adolescents in the Pacific. PLoS One. (2019) 14:e0214420. doi: 10.1371/journal.pone.0214420, PMID: 30901361 PMC6430393

[21] Scalese M Cerrai S Biagioni S Benedetti E Bastiani L Potente R. Trends in energy drink and combined alcohol and energy drinks consumption among Italian high school students, 2008–2019. Drug Alcohol Depend. (2021) 228:109061. doi: 10.1016/j.drugalcdep.2021.109061, PMID: 34601280

[22] Scalese M Denoth F Siciliano V Bastiani L Cotichini R Cutilli A. Energy drink and alcohol mixed energy drink use among high school adolescents: association with risk taking behavior, social characteristics. Addict Behav. (2017) 72:93–9. doi: 10.1016/j.addbeh.2017.03.016, PMID: 28388494

[23] Trapp G Hurworth M Christian H Bromberg M Howard J McStay C. Prevalence and pattern of energy drink intake among Australian adolescents. J Hum Nutr Diet. (2021) 34:300–4. doi: 10.1111/jhn.12789, PMID: 32827226

[24] Galimov A Hanewinkel R Hansen J Unger JB Sussman S Morgenstern M. Energy drink consumption among German adolescents: prevalence, correlates, and predictors of initiation. Appetite. (2019) 139:172–9. doi: 10.1016/j.appet.2019.04.016, PMID: 31047938

[25] Azagba S Langille D Asbridge M. An emerging adolescent health risk: caffeinated energy drink consumption patterns among high school students. Prev Med. (2014) 62:54–9. doi: 10.1016/j.ypmed.2014.01.019, PMID: 24502849

[26] Carsi Kuhangana T Muta Musambo T Pyana Kitenge J Kayembe-Kitenge T Kazadi Ngoy A Musa Obadia P. Energy drink consumption among adolescents attending schools in Lubumbashi, Democratic Republic of Congo. Int J Environ Res Public Health. (2021) 18:7617. doi: 10.3390/ijerph18147617, PMID: 34300068 PMC8304143

[27] Miller KE Dermen KH Lucke JF. Caffeinated energy drink use by US adolescents aged 13–17: a national profile. Psychol Addict Behav. (2018) 32:647–59. doi: 10.1037/adb0000389, PMID: 30124307 PMC6136946

[28] Faris MA Epuru S Al-Shimmari S Al-Shimmari E. Alarming high levels of energy drinks consumption among school children in hail, northern of Saudi Arabia. Int J Child Health Nutr. (2015) 4:1–13. doi: 10.6000/1929-4247.2015.04.01.1

[29] Musaiger A Zagzoog N. Knowledge, attitudes and practices toward energy drinks among adolescents in Saudi Arabia. Glob J Health Sci. (2013) 6:42–6. doi: 10.5539/gjhs.v6n2p42, PMID: 24576364 PMC4825248

[30] Almulla AA Faris MAE. Energy drinks consumption is associated with reduced sleep duration and increased energy-dense fast foods consumption among school students: a Cross-sectional study. Asia Pac J Public Health. (2020) 32:266–73. doi: 10.1177/1010539520931351, PMID: 32508133

[31] Von Elm E Altman DG Egger M Pocock SJ Gøtzsche PC Vandenbroucke JP. The strengthening the reporting of observational studies in epidemiology (STROBE) statement: guidelines for reporting observational studies. Lancet. (2007) 370:1453–7. doi: 10.1016/S0140-6736(07)61602-X, PMID: 18064739

[32] Nuss T Morley B Scully M Wakefield M. Energy drink consumption among Australian adolescents associated with a cluster of unhealthy dietary behaviours and short sleep duration. Nutr J. (2021) 20:64. doi: 10.1186/s12937-021-00719-z, PMID: 34225738 PMC8259213

[33] UAE. *Supermarkets take action on energy drink sales*. (n.d.). Available at: https://www.thenationalnews.com/uae/uae-supermarkets-take-action-on-energy-drink-sales-1.698238.

[34] Dubai Schools Ban Energy Drinks. *Why such beverages are harmful for children*. (n.d.). Available at: https://www.khaleejtimes.com/uae/dubai-school-bans-energy-drinks-why-such-beverages-are-harmful-for-children.

[35] The National. *Doctors and parents back Dubai school's prime energy drink warning*. (n.d.). Available at: https://www.thenationalnews.com/uae/2023/05/28/doctors-and-parents-back-dubai-schools-prime-energy-drink-warning/.

[36] Dillon P Kelpin S Kendler K Thacker L Dick D Svikis D. Gender differences in any-source caffeine and energy drink use and associated adverse health behaviors. J Caffeine Adenosine Res. (2019) 9:12–9. doi: 10.1089/caff.2018.0008, PMID: 30944911 PMC6444914

[37] Lebacq T Desnouck V Dujeu M Holmberg E Pedroni C Castetbon K. Determinants of energy drink consumption in adolescents: identification of sex-specific patterns. Public Health. (2020) 185:182–8. doi: 10.1016/j.puhe.2020.05.040, PMID: 32645505

[38] Emond JA Gilbert-Diamond D Tanski SE Sargent JD. Energy drink consumption and the risk of alcohol use disorder among a National Sample of adolescents and young adults. J Pediatr. (2014) 165:1194–200. doi: 10.1016/j.jpeds.2014.08.050, PMID: 25294603 PMC4252708

[39] Evren C Evren B. Energy-drink consumption and its relationship with substance use and sensation seeking among 10th grade students in Istanbul. Asian J Psychiatr. (2015) 15:44–50. doi: 10.1016/j.ajp.2015.05.001, PMID: 26006774

[40] Bryant Ludden A Wolfson AR. Understanding adolescent caffeine use: connecting use patterns with expectancies, reasons, and sleep. Health Educ Behav. (2009) 37:330–42. doi: 10.1177/1090198109341783, PMID: 19858312

[41] Chu JY Porche MV Tolman DL. The adolescent masculinity ideology in relationships scale: development and validation of a new measure for boys. Men Masculinities. (2005) 8:93–115. doi: 10.1177/1097184X03257453

[42] Pletzer B Petasis O Ortner TM Cahill L. Interactive effects of culture and sex hormones on the sex role self-concept. Front Neurosci. (2015) 9:240. doi: 10.3389/fnins.2015.00240, PMID: 26236181 PMC4500910

[43] Levant RF Parent MC McCurdy ER Bradstreet TC. Moderated mediation of the relationships between masculinity ideology, outcome expectations, and energy drink use. Health Psychol. (2015) 34:1100–6. doi: 10.1037/hea0000214, PMID: 25730610

[44] Rawlins E Baker G Maynard M Harding S. Perceptions of healthy eating and physical activity in an ethnically diverse sample of young children and their parents: the DEAL prevention of obesity study. J Hum Nutr Diet. (2013) 26:132–44. doi: 10.1111/j.1365-277X.2012.01280.x, PMID: 22827466 PMC3618369

[45] Liu KSN Chen JY Ng MYC Yeung MHY Bedford LE Lam CLK. How does the family influence adolescent eating habits in terms of knowledge, attitudes and practices? A global systematic review of qualitative studies. Nutrients. (2021) 13:13. doi: 10.3390/nu13113717, PMID: 34835973 PMC8624651

[46] Sampasa-Kanyinga H Masengo L Hamilton HA Chaput J-P. Energy drink consumption and substance use among middle and high school students. Int J Environ Res Public Health. (2020) 17:3110. doi: 10.3390/ijerph17093110, PMID: 32365667 PMC7246708

[47] Berenbaum SA Beltz AM. How early hormones shape gender development. Curr Opin Behav Sci. (2016) 7:53–60. doi: 10.1016/j.cobeha.2015.11.011, PMID: 26688827 PMC4681519

[48] McLellan TM Caldwell JA Lieberman HR. A review of caffeine’s effects on cognitive, physical and occupational performance. Neurosci Biobehav Rev. (2016) 71:294–312. doi: 10.1016/j.neubiorev.2016.09.001, PMID: 27612937

[49] Champlin SE Pasch KE Perry CL. Is the consumption of energy drinks associated with academic achievement among college students? J Prim Prev. (2016) 37:345–59. doi: 10.1007/s10935-016-0437-4, PMID: 27236788

[50] Khan MS Nisar N Arsalan S Naqvi A Nawab F. Caffeine consumption and academic performance among medical students of Dow University of health science (DUHS), Karachi, Pakistan. Ann Abbasi Shaheed Hosp Karachi Med Dent Coll. (2017) 22:179–84. doi: 10.58397/ashkmdc.v22i3.126

[51] Ghozayel M Ghaddar A Farhat G Nasreddine L Kara J Jomaa L. Energy drinks consumption and perceptions among university students in Beirut, Lebanon: a mixed methods approach. PLoS One. (2020) 15:e0232199. doi: 10.1371/journal.pone.0232199, PMID: 32353017 PMC7192412

[52] Kaldenbach S Strand TA Solvik BS Holten-Andersen M. Social determinants and changes in energy drink consumption among adolescents in Norway, 2017–2019: a cross-sectional study. BMJ Open. (2021) 11:e049284. doi: 10.1136/bmjopen-2021-049284, PMID: 34417216 PMC8381306

[53] Thomas PA Liu H Umberson D. Family relationships and well-being. Innov Aging. (2017) 1:igx025. doi: 10.1093/geroni/igx025, PMID: 29795792 PMC5954612

[54] Larson N DeWolfe J Story M Neumark-Sztainer D. Adolescent consumption of sports and energy drinks: linkages to higher physical activity, unhealthy beverage patterns, cigarette smoking, and screen media use. J Nutr Educ Behav. (2014) 46:181–7. doi: 10.1016/j.jneb.2014.02.008, PMID: 24809865 PMC4023868

[55] Degirmenci N Fossum IN Strand TA Vaktskjold A Holten-Andersen MN. Consumption of energy drinks among adolescents in Norway: a cross-sectional study. BMC Public Health. (2018) 18:1391. doi: 10.1186/s12889-018-6236-5, PMID: 30567510 PMC6299924

[56] Bradbury KM Turel O Morrison KM. Electronic device use and beverage related sugar and caffeine intake in US adolescents. PLoS One. (2019) 14:e0223912. doi: 10.1371/journal.pone.0223912, PMID: 31639162 PMC6805001

[57] Sampasa-Kanyinga H Hamilton HA Chaput J-P. Sleep duration and consumption of sugar-sweetened beverages and energy drinks among adolescents. Nutrition. (2018) 48:77–81. doi: 10.1016/j.nut.2017.11.013, PMID: 29469025

[58] Galemore CA. Sports drinks and energy drinks for children and adolescents–are they appropriate?: a summary of the clinical report. NASN Sch Nurse. (2011) 26:320–1. doi: 10.1177/1942602X11417310, PMID: 21957570

[59] Espinosa Jovel CA Sobrino Mejía FE. Caffeine and headache: specific remarks. Neurología. (2017) 32:394–8. doi: 10.1016/j.nrleng.2014.12.022, PMID: 25728949

[60] Garg A Rodriguez A Lewis JT Bansal R Brahmbhatt B. Energy drinks: a reversible risk factor for atrophic gastritis and gastric intestinal metaplasia. Cureus. (2020) 12:e12298. doi: 10.7759/cureus.12298, PMID: 33520500 PMC7834582

[61] Alsunni AA. Energy drink consumption: beneficial and adverse health effects. Int J Health Sci. (2015) 9:459–65. doi: 10.12816/0031237, PMID: 26715927 PMC4682602

[62] Halldorsson TI Kristjansson AL Thorisdottir I Oddsdóttir C Sveinbjörnsson J Benediktsson R. H G: caffeine exposure from beverages and its association with self-reported sleep duration and quality in a large sample of Icelandic adolescents. Food Chem Toxicol. (2021) 157:112549. doi: 10.1016/j.fct.2021.112549, PMID: 34509583

[63] Veselska ZD Husarova D Kosticova M. Energy drinks consumption associated with emotional and Behavioural problems via lack of sleep and skipped breakfast among adolescents. Int J Environ Res Public Health. (2021) 18:6055. doi: 10.3390/ijerph18116055, PMID: 34199877 PMC8200076

[64] Buysse DJ Reynolds CF 3rd Monk TH Berman SR Kupfer DJ. The Pittsburgh sleep quality index: a new instrument for psychiatric practice and research. Psychiatry Res. (1989) 28:193–213. doi: 10.1016/0165-1781(89)90047-4, PMID: 2748771

[65] How blue light affects kids’ sleep?. (n.d.). Available at: https://www.sleepfoundation.org/children-and-sleep/how-blue-light-affects-kids-sleep#:~:text=View%20Source%20and%20duration.,than%20we%20should%20at%20bedtime.

[66] Faris MAE Jahrami H Al-Hilali MM Chehyber NJ Ali SO Shahda SD. Energy drink consumption is associated with reduced sleep quality among college students: a cross-sectional study. Nutr Diet. (2017) 74:268–74. doi: 10.1111/1747-0080.12289, PMID: 28731611

[67] Lohsoonthorn V Khidir H Casillas G Lertmaharit S Tadesse MG Pensuksan WC. Sleep quality and sleep patterns in relation to consumption of energy drinks, caffeinated beverages, and other stimulants among Thai college students. Sleep Breath. (2013) 17:1017–28. doi: 10.1007/s11325-012-0792-1, PMID: 23239460 PMC3621002

[68] Richards G Smith AP. Breakfast and energy drink consumption in secondary school children: breakfast omission, in isolation or in combination with frequent energy drink use, is associated with stress, anxiety, and depression Cross-Sectionally, but not at 6-month follow-up. Front Psychol. (2016) 7:106. doi: 10.3389/fpsyg.2016.00106, PMID: 26903914 PMC4746319

[69] Alafif N Al-Rashed A Altowairqi K Muharraq A. Prevalence of energy drink consumption and association with dietary habits among governmental university students in Riyadh. Saudi J Biol Sci. (2021) 28:4511–5. doi: 10.1016/j.sjbs.2021.04.050, PMID: 34354437 PMC8324967

[70] Marinoni M Parpinel M Gasparini A Ferraroni M Edefonti V. Psychological and socio-educational correlates of energy drink consumption in children and adolescents: a systematic review. Eur J Pediatr. (2022) 181:889–901. doi: 10.1007/s00431-021-04321-7, PMID: 34825275

[71] Al Gharaibeh F Mahmud A Islam MR. Community initiatives against Covid-19 in the United Arab Emirates. Community Dev J. (2022) 57:404–10. doi: 10.1093/cdj/bsab045

[72] Islam MR Al Gharaibeh F Azman A Hashim IH Islam MR Rahman A. Social behavior practices for child protection and well-being among low-income urban households in Bangladesh. Asian Soc Work Policy Rev. (2023) 17:39–51. doi: 10.1111/aswp.12270

